# Resting-State EEG Source Localization and Functional Connectivity in Schizophrenia-Like Psychosis of Epilepsy

**DOI:** 10.1371/journal.pone.0027863

**Published:** 2011-11-18

**Authors:** Leonides Canuet, Ryouhei Ishii, Roberto D. Pascual-Marqui, Masao Iwase, Ryu Kurimoto, Yasunori Aoki, Shunichiro Ikeda, Hidetoshi Takahashi, Takayuki Nakahachi, Masatoshi Takeda

**Affiliations:** 1 Department of Psychiatry and Clinical Neuroscience, Osaka University Graduate School of Medicine, Suita City, Osaka, Japan; 2 The KEY Institute for Brain-Mind Research, University Hospital of Psychiatry, Zurich, Switzerland; 3 Department of Neuropsychiatry, Kansai Medical University, Osaka, Japan; University Medical Center Groningen UMCG, The Netherlands

## Abstract

**Background:**

It is unclear whether, like in schizophrenia, psychosis-related disruption in connectivity between certain regions, as an index of intrinsic functional disintegration, occurs in schizophrenia-like psychosis of epilepsy (SLPE). In this study, we sought to determine abnormal patterns of resting-state EEG oscillations and functional connectivity in patients with SLPE, compared with nonpsychotic epilepsy patients, and to assess correlations with psychopathological deficits.

**Methodology/Principal Findings:**

Resting EEG was recorded in 21 patients with focal epilepsy and SLPE and in 21 clinically-matched non-psychotic epilepsy controls. Source current density and functional connectivity were determined using eLORETA software. For connectivity analysis, a novel nonlinear connectivity measure called “*lagged phase synchronization*” was used. We found increased theta oscillations in regions involved in the default mode network (DMN), namely the medial and lateral parietal cortex bilaterally in the psychotic patients relative to their nonpsychotic counterparts. In addition, patients with psychosis had increased beta temporo-prefrontal connectivity in the hemisphere with predominant seizure focus. This functional connectivity in temporo-prefrontal circuits correlated with positive symptoms. Additionally, there was increased interhemispheric phase synchronization between the auditory cortex of the affected temporal lobe and the Broca's area correlating with auditory hallucination scores.

**Conclusions/Significance:**

In addition to dysfunction of parietal regions that are part of the DMN, resting-state disrupted connectivity of the medial temporal cortex with prefrontal areas that are either involved in the DMN or implicated in psychopathological dysfunction may be critical to schizophrenia-like psychosis, especially in individuals with temporal lobe epilepsy. This suggests that DMN deficits might be a core neurobiological feature of the disorder, and that abnormalities in theta oscillations and beta phase synchronization represent the underlying neural activity.

## Introduction

There is substantial evidence indicating that several brain regions are dysfunctional in schizophrenia and other psychotic disorders, both during rest and cognitive task performance [1], [2]. However, this kind of approach, characterizing brain activity purely in terms of anatomically segregated responses, is not sufficient to explain the pathophysiology of such complex disorders. For a better understanding of the abnormalities underlying the clinical deficits in schizophrenia, recent studies have looked at structural and functional connectivity between brain regions [3]–[7]. These studies found aberrant connectivity patterns in distributed brain networks that often correlate with cognitive deficits and core symptoms of the disease. This has led to the suggestion that schizophrenia is best characterized as a disruption of functional integration of neural systems rather than as regionally localized abnormalities, thus supporting the notion of schizophrenia as a “disconnection syndrome” [8].

Schizophrenia-like psychosis of epilepsy (SLPE) is a neuropsychiatric disorder that clinically closely resembles schizophrenia with a typical presentation as a paranoid-hallucinatory syndrome along with other psychopathological disturbances and cognitive deficits. However, compared with schizophrenia, SLPE distinguishes itself by a relative absence of negative symptoms, lesser severity, and better premorbid as well as long-term functioning [9], [10]. Several neuroimaging studies looking at localized structural and functional abnormalities at rest and during cognitive tasks in SLPE have revealed subtle or prominent cortical pathology and cerebral dysfunction mainly localized to temporal and frontal regions [11]–[14]. For instance, in a recent study using magnetoencephalography, we found working memory-related dysfunction localized to the prefrontal and left temporal cortex in patients with SLPE [15], with the activity in the prefrontal cortex correlating with psychopathological measures [16]. Others have noticed white matter structural abnormalities in SLPE using diffusion tensor imaging [17]. This suggests that like in schizophrenia, in addition to gray matter deficits and cortical dysfunction, white matter pathology and disrupted connections between brain areas may also play a role in the pathophysiology of SLPE. In support to this view, there is evidence that a number of diseases of the white matter such as leukodystrophies, neoplasms, inflammatory diseases, and callosal anomalies can present as a schizophrenia-like disorder [18]. However, to date, no attempt has been made to determine abnormal patterns of functional connectivity in SLPE either in resting state or during performance of cognitive tasks.

Recently, the ongoing intrinsic activity in the human brain during resting state has attracted considerable attention in neuroscience. The brain resting state is thought to be an energetically costly condition characterized by a rich neural activity and long-range interneuron connections in specific brain circuits (e.g., the default mode network or DMN) that are temporally interrupted or attenuated during performance of sensorimotor or cognitively-demanding tasks [19], [20]. This intrinsic functional organization during rest allows the brain to allocate resources and ready itself for changes in internal and external environments. Therefore, the investigation of functional connectivity during resting state rather than during a particular task may reveal an intrinsic functional disintegration between brain regions in SLPE.

Functional connectivity refers to the temporal synchrony or correlation between signals of two or more spatially separated regions as an index of functional integration between neural populations [21]–[24]. In psychotic disorders, functional connectivity has often been examined using functional MRI due to the high spatial resolution of this technique that allows for an accurate localization of brain regions with abnormal functional interactions [4]. However, unlike neurophysiological techniques including EEG, MRI has a low temporal resolution, and is not able to measure neural activity directly but instead relies on the hemodynamic changes that may occur in response to neural activity [25]. Because EEG time-series data directly relate to dynamic postsynaptic activity in the cerebral cortex with a high temporal resolution, EEG is suitable to visualize synchronization across frequency bands in large-scale functional networks. Despite these advantages, EEG suffers from the problem of volume conduction or common sources, which gives rise to spurious correlations between time series recorded from nearby electrodes [26]. Although several approaches have been proposed to overcome this limitation [26], [27], finding reliable measures of physiological functional connectivity remains challenging.

Exact low resolution brain electromagnetic tomography or eLORETA is a linear inverse solution method that can be applied to high time resolution, scalp EEG recordings to compute the three-dimensional distribution of electric cortical activity at any number of locations in the brain with no localization error to point sources under ideal (noise-free) conditions [28]–[30]. In addition, measures of “similarity” between any pair of intracranial signals can be obtained as an index of physiological “*lagged connectivity*” between the respective sites. This novel connectivity measure is resistant to non-physiological artifacts, in particular volume conduction and low spatial resolution that usually affect other connectivity indices [26], [27]. Furthermore, it can be applied to filtered data, thus giving a frequency decomposition of brain connectivity [30]–[32].

In this study, we sought to determine abnormal patterns of resting-state EEG oscillatory activity and functional connectivity in patients with SLPE using lagged phase synchronization, a novel nonlinear connectivity measure implemented in the free academic eLORETA software. We also aimed at assessing whether abnormalities in functional connectivity in SLPE correlate with psychopathological disturbance. Based on previous findings in schizophrenia, our working hypothesis was that during resting state, in addition to regional cortical dysfunction, disruption in functional connectivity may also occur in patients with SLPE affecting areas of the DMN as well as cortical regions critical to psychosis, and that some altered connections between brain areas may correlate with psychopathology measures.

## Methods

### Definition of Schizophrenia-like Psychosis of Epilepsy

Schizophrenia-like psychosis was diagnosed by qualified neuropsychiatrists, including the authors of this study, based on DSM-IV criteria for schizophreniform disorders [33]. According to these criteria, patients present with two or more of the following symptoms: 1) delusions, 2) hallucinations, 3) disorganized speech, 4) disorganized or catatonic behavior, and 5) negative symptoms, with at least one episode lasting for 1 month or more, in the absence of a schizoaffective disorder diagnosis or mood disorder with psychotic features. In addition, the psychosis developed after the onset of epilepsy, all psychotic episodes lasted more than 24 h and occurred interictally–during seizure-free periods or between habitual seizures–in a state of full consciousness [16], [34]–[36]. Ictal psychotic phenomenon, postictal psychosis (psychotic events occurring within seven days of a seizure or clusters of seizures), and brief interictal psychosis (all episodes resolve in less than 1 month) were excluded.

### Subjects

Twenty-one patients with focal epilepsy and SLPE (age 34.6±10.1 years; eleven men) and 21 non-psychotic epilepsy (nPE) controls (age 34.5±9.0 years; twelve men) matched for age, type of epilepsy (i.e., temporal or frontal lobe epilepsy) and laterality of seizure focus participated in this study. These patients were ascertained from an epilepsy database in Osaka University Hospital [37]. Epilepsy classification followed the standards set by the International League Against Epilepsy [38]. Epilepsy diagnosis and seizure focus localization were determined on the basis of seizure semiology, findings from ictal/interictal EEG, and coregistration of spatially filtered MEG data with MRI results [39]–[41]. All patients with SLPE had chronic interictal psychosis with onset 15.2±9.4 years after the epilepsy. The groups did not differ in demographic or clinical characteristics (Table 1). Patients with gross organic lesions and an IQ score below 70, as indicated by the Wechsler Adult Intelligence Scale-Revised (WAIS-R) were excluded, as were those with age greater than 55 or history of drug/alcohol abuse. Most patients were on carbamazepine therapy, matched for plasma levels of the drug (SLPE: 7.2±3.6 µg/ml, nPE: 7.8±3.1 µg/ml, p = 0.70), and some were taking valproic acid; two patients in the SLPE group were antiepileptic-drug free. Most patients with psychosis were on regiments involving neuroleptic drugs, mostly atypical antipsychotics (i.e., aripiprazole, risperidone, olanzapine) with a chlorpromazine equivalent dose of 203.7±308.4 mg/day; nine patients were antipsychotic-drug free. None of the patients were taking sedatives or antidepressants at the time of the study. All patients provided written informed consent, and ethical approval was obtained from the ethics committee of Osaka University Hospital.

**Table 1:** Demographic and clinical characteristics.

Characteristics	SLPEn = 21	nPEn = 21	t	p
Age, years	34.6±10.1	34.5±9.0	0.05	0.96
Sex, n (F/M)	10/11	9/12	–	–
Education, years	13.1±2.3	14.7±2.0	−2.38	0.022
WAIS-R IQ	83.2±15.3	96.9±16.4	−2.78	0.009
Epilepsy onset, years	14.4±13.2	17.1±11.8	−0.65	0.51
Epilepsy duration, years	20.2±10.4	17.2±9.4	0.97	0.33
Seizure frequency/year	10.5±18.6	13.3±26.9	−0.38	0.70
Epilepsy type, n (TLE/FLE)	19/2	19/2		
Psychosis onset, years	28.8±11.3			
Psychosis duration, years	5.0±4.3			
BPRS total score	49.1±11.4			
Positive Symptoms	12.0±3.1			
Negative Symptoms	10.5±4.0			
Disorganization	8.2±2.5			
Affect domain	13.7±3.8			

Data are mean ± SD unless otherwise noted. SLPE, schizophrenia-like psychosis of epilepsy; nPE, nonpychotic epilepsy; WAIS-R, Wechsler Adult Intelligent Scale-Revised; BPRS, Brief Psychiatric Rating Scale; TLE, temporal lobe epilepsy; FLE, frontal lobe epilepsy.

### Psychopathological Assessment

Psychopathology was assessed in patients with SLPE using the Brief Psychiatric Rating Scale (BPRS) on the day of the EEG recordings. A 4-factor structure of clinical symptoms, reported to fit the data better than the typical 5-factor BPRS model for schizophrenia, was used for analyses [42]. Symptoms are grouped into four major syndromes: ‘thought disturbance’ (positive symptoms), ‘anergia’ (negative symptoms), ‘disorganization’ and ‘affect’ domain. *Positive symptoms* are determined by summing up scores on grandiosity, suspiciousness, hallucinatory behavior and delusion/unusual-thought content items. *Negative symptoms* include emotional withdrawal, motor retardation, uncooperativeness and blunted affect, while *disorganization* was defined by conceptual disorganization, tension and mannerisms/posturing, and the *affect* domain by somatic concern, anxiety, guilt feelings, depressed mood, and hostility. Since hallucinations and delusions are the core psychiatric symptoms of schizophrenia-like disorders, we also used the raw scores of 1) hallucinatory behavior and 2) unusual-thought content (delusions) items of the BPRS scale for analyses of the data.

### EEG Recordings and Data Acquisition

Digital 19-channel EEG was recorded using a Nihon-Kohden (Inc., Tokyo, Japan) system with the electrodes positioned according to the International 10–20 system (i.e., Fp1, Fp2, F3, F4, C3, C4, P3, P4, O1, O2, F7, F8, T7, T8, P7, P8, Fz, Cz, Pz). The EEG activity was acquired using a linked ears reference, sampled at 500 Hz, and filtered offline between 1 and 30 Hz. Impedance was kept below 5 kΩ. Vigilance-controlled recordings were made according to usual clinical standards, including a 10-min resting eyes-closed state, 3 min of alternate 10 s-eyes open/eyes closed conditions, 3 min of hyperventilation, 1 min of recovery (post-hyperventilation), and photic stimulation. Analysis was circumscribed to the resting, awake, eyes-closed state. Brain Electrical Source Analysis (BESA) software (www.besa.de) was used for visual inspection of the EEG recordings and manual selection of samples. For each subject, 15 non-overlapping, 2-s artifact-and spike-free segments were randomly selected. We carefully avoided, particularly epochs containing ocular movements (present in up to 40% of the 10-min eyes-closed resting state recording, especially in many patients with psychosis), muscle or cardiac contamination, drowsiness signs (i.e., emergence of slow wave activity with suppression of alpha rhythm, frequently seen in the last 1–3 min of the eyes-closed state recording in several patients), spikes or sharp waves, and even small baseline shifts so that reliable estimates of brain function in the awake resting-state could be obtained. Further analyses were performed using the LORETA-KEY software package as provided at www.uzh.ch/keyinst/LORETA.html.

### EEG-Source Localization Analysis

To compute the intracortical distribution of the electric activity from the surface EEG data, we used eLORETA [28]–[30]. The eLORETA method is a discrete, three-dimensional (3D) distributed, linear, weighted minimum norm inverse solution. The particular weights used in eLORETA endow the tomography with the property of exact localization to test point sources, yielding images of current density with exact localization albeit with low spatial resolution (i.e. neighboring neuronal sources will be highly correlated). A further property of eLORETA is that it has no localization bias even in the presence of structured noise [28]. In this sense, eLORETA is an improvement over previously related tomographies LORETA [43] and the standardized version sLORETA [44].

In the current eLORETA implementation, the solution space was restricted to the cortical gray matter, corresponding to 6239 voxels at 5×5×5 mm spatial resolution. The Montreal Neurologic Institute average MRI brain (MNI152) [45] was used as a realistic head model for which the lead field was computed [46]. Validation for the new improved eLORETA tomography rests upon the abundant published validation for the previous LORETA and sLORETA methods. For instance, excellent localization agreement has been reported in multimodal imaging studies with functional MRI [47], [48], structural MRI [49], and PET [50]–[52]. Further validation based on accepting as “ground truth” the information provided by intracranial recordings in humans has been reported in a number of articles [53], [54].

Selected artifact-free EEG segments were used for calculating the eLORETA intracranial spectral density with a resolution of 1 Hz, from 1 to 30 Hz. eLORETA functional images of spectral density were computed for six frequency bands: delta (1.5–4 Hz), theta (4–8 Hz), alpha1 (8–10 Hz), alpha2 (10–13 Hz), beta1 (13–21 Hz) and beta2 (21–30 Hz).

The difference in source localization of cortical oscillations between groups in each frequency band was assessed by voxel-by-voxel independent sample *F-ratio*-tests, based on eLORETA log-transformed current density power. In the resulting statistical three-dimensional images, cortical voxels showing significant differences were identified by a nonparametric approach (statistical nonparametric mapping or SnPM) via randomizations. This randomization strategy [55] determined the critical probability threshold values for the actually observed *t*-values with correction for multiple comparisons across all voxels and all frequencies. A total of 5000 permutations were used to determine the significance for each randomization test. The use of statistical nonparametric maps applied to LORETA images has been validated in several studies [56]–[58]. Although these statistical tests focused mainly on signal strength for single voxels (referred to as ‘single voxel statistics’), a second nonparametric analysis assessed the significance of activity based on its spatial extent, obtaining clusters of supra-threshold voxels (referred to as “cluster statistics”) [59]. The LORETA method does not need any “distributional assumptions” yielding an adjusted t-critical value that is effective for controlling Type I error [60].

### Functional Connectivity Analysis

For functional connectivity analysis, we used a “whole-brain Brodmann areas (BAs)” approach, selecting all 42 BAs in each hemisphere as regions of interest (ROI). The anatomical definitions of BAs provided by eLORETA software package are based on the Talairach Daemon (http://www.talairach.org/). For the analysis of connectivity between pairs of BAs, a method using a single voxel at the centroid of each BA was chosen. This procedure is justified because eLORETA has a low spatial resolution, which makes it unable to separate two closely spaced sources, and additionally, the single centroid voxel (the closest to the center of the BA mass) is an excellent representative of the corresponding BA.

A novel index of physiological lagged connectivity, namely lagged phase synchronization was used as measure of functional connectivity between any pair of BAs [30]–[32]. Lagged phase synchronization measures the similarity (a corrected phase synchrony value) between signals in the frequency domain based on normalized (unit module) Fourier transforms; thus it is related to nonlinear functional connectivity. This lagged connectivity measure is accurately corrected as it represents the connectivity between two signals after the instantaneous zero-lag contribution has been excluded. Such a correction is necessary when using scalp EEG signals or estimated intracranial signals (EEG tomography) because zero-lag connectivity in a given frequency band is often due to non-physiological effects or intrinsic physics artifacts, in particular volume conduction and low spatial resolution that usually affect other connectivity indices [26], [27]. Thus, this measure is thought to contain only physiological connectivity information.

The classical total “squared” phase synchronization, which is highly contaminated by the instantaneous artifactual component, is defined as:



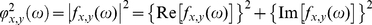
(1)


with:



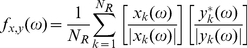
(2)


where 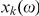 and 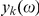 denote the discrete Fourier transforms of the two signals of interest *x* and *y* at frequency 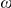 for the *k*-th EEG segment or epoch 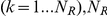 being the number of epochs. Re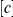 and Im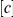 denote the real and imaginary parts of a complex number *c*; 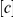 denotes the modulus of *c*; and the superscript “*”, a complex conjugate. The instantaneous (zero-lag) connectivity component is closely related to the real part of the phase synchronization. Lagged phase synchronization, which statistically partials out the instantaneous component of the total connectivity, is defined as:



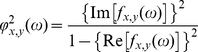
(3)


Ideally, any change in the instantaneous component should have little effect on the lagged or on the imaginary connectivity. However, when a lagged connection exists, the imaginary part of connectivity measures such as coherence [27] fails to detect it by tending to zero if the instantaneous component is large. This is not the case for lagged phase synchronization in Eq. 3 which asymptotically tends to a nonzero value, detecting the presence of a physiological lagged connection. Details on eLORETA connectivity algorithm can be found in recent reports by Pascual-Marqui *et al.* [30], [31].

For comparison of eLORETA functional connectivity between groups (psychotic vs. non-psychotic patients) in each frequency band, independent sample *t*-tests and randomizations were performed [55]. As mentioned above, this randomization strategy allows for correction for multiple comparisons without the need to rely on Gaussianity.

To assess the correlation between eLORETA connectivity measures and external clinical variables, in particular psychopathology measures, the product-moment correlations of lagged phase synchronization values between all pairs of BAs and BPRS scores (i.e., positive symptoms, negative symptoms, disorganization, affect domain, and BPRS total score) were computed, and randomization determined the critical probability threshold values for the actually observed *r*-values with correction for multiple testing. Overall, 5166 tests were performed by eLORETA to compare all connections between 42 BAs (861 connections) for each of the six frequency bands (861×6 = 5166), and to correlate these functional connections with an external clinical variable (i.e., BPRS symptom scores) for each analysis, with the nonparametric randomization procedure yielding *t* and *r*-critical values adjusted for multiple comparisons. Chi square and t-tests for analysis of demographic and clinical data were carried out using SPSS software version 17 (SPSS Inc. Chicago, IL).

## Results

The demographic and clinical characteristics of our sample are given in Tab 1. There were no differences in age, sex and epilepsy-related features between groups. The vast majority of the patients had temporal lobe epilepsy (*n* = 38/42), and near two-thirds of them (63.16%) had been diagnosed with a right seizure focus (left, *n* = 14; right, *n* = 24). Although all patients had an IQ of 75 or higher, those in the psychotic group had lower IQ and educational level compared with their nonpsychotic counterparts. Psychopathology scores in the psychotic groups are also shown in Tab 1. All patients recruited for this study who had hallucinatory behavior reported verbal auditory type of hallucinations.

### Source Localization

**Figure 1:**
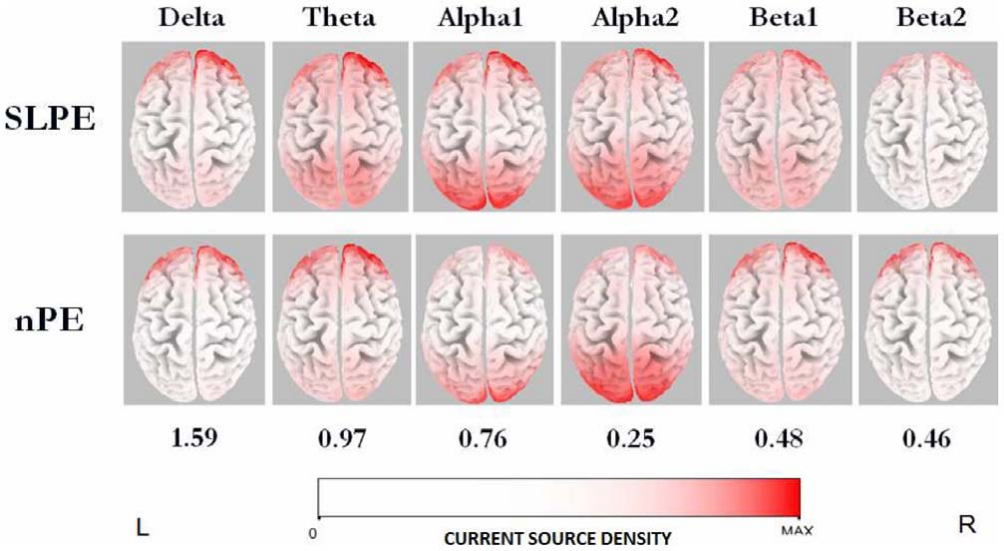
Averaged eLORETA solutions (current density at cortical voxels) of EEG sources for each frequency band. The maximum current density values for each frequency are given below the corresponding column. SLPE, schizophrenia-like psychosis of epilepsy; nPE, nonpsychotic epilepsy; L, left; R, right.

**Figure 2:**
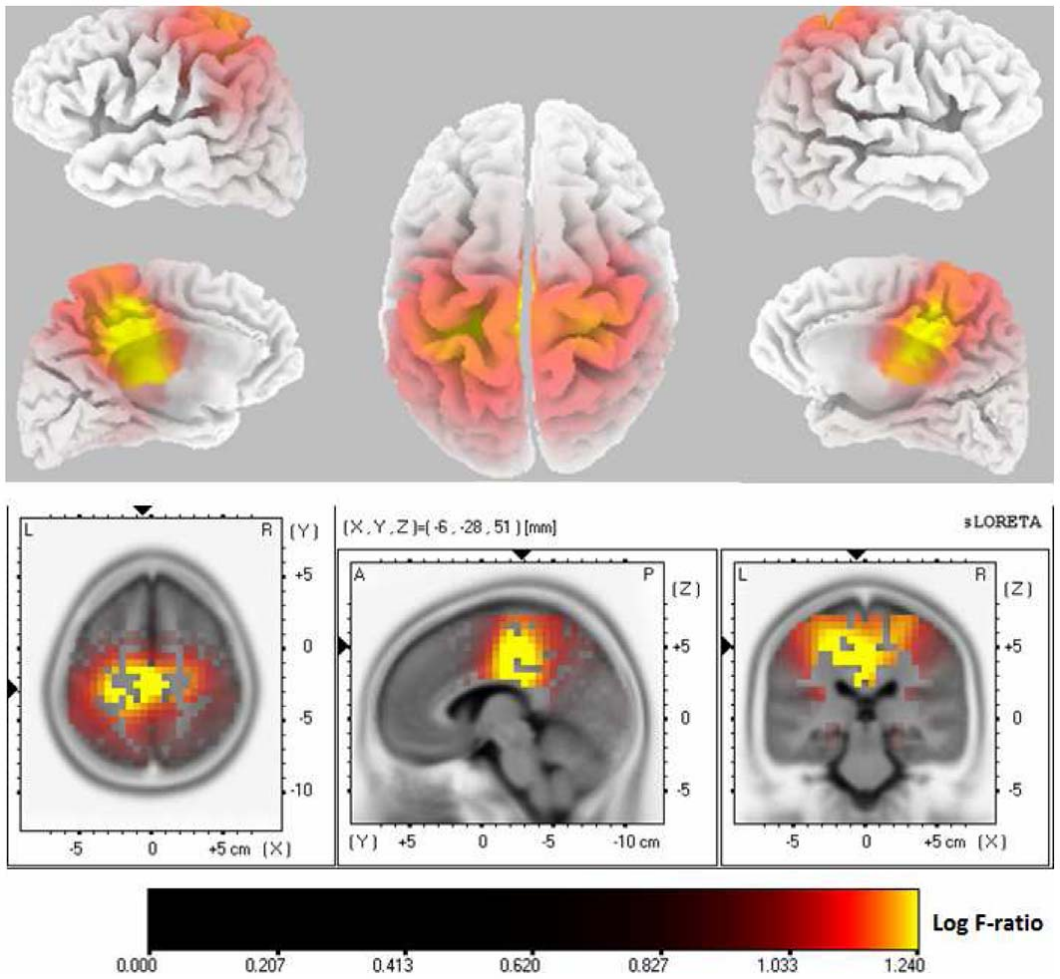
eLORETA statistical maps of theta oscillations. Colored areas represent the spatial extent of voxels with significant difference (red-coded for p<0.05; yellow-coded for p<0.01, corrected for multiple testing) in source current density in psychotic vs. nonpsychotic epilepsy patients. Significant results are projected onto a fiducial cortical surface (top panel) and a brain MRI template (bottom panel). The MRI slices are located at the MNI-space coordinates indicated in the figure that correspond to the voxel of highest significance (i.e., left posterior cingulate/precuneus cortex). The color scale represents log F-ratio values (threshold: log-F = 1.03, p<0.05). L, left; R, right; A, anterior; P, posterior.

Fig. 1 shows the averaged eLORETA solutions. Electric neuronal activity was higher in patients with SLPE compared to nonpsychotic patients for the slower frequency bands. Higher current density maxima were found particularly in delta (SLPE: 1.59±1.04, nPE: 1.35±0.98), theta (SLPE: 0.97±0.88, nPE: 0.50±0.24) as well as in the lower alpha band (SLPE: 0.76±1.13, nPE: 0.66±1.07). There was a similar cortical distribution of maximal activity across groups; delta and theta bands had maximum values in the prefrontal cortex, whereas alpha1 activity was maximal in occipital regions. Theta oscillations were also observed over the parietal cortex in patients with SLPE. Pronounced slow oscillatory activity was not seen in temporal sites. The nonpsychotic group exhibited slightly higher source current density maxima for the rest of the frequency bands, with alpha2 (SLPE: 0.20±0.14, nPE: 0.25±0.10) maxima in the occipital cortex, and beta1 (SLPE: 0.41±0.25, nPE: 0.48±0.29) and beta2 (SLPE: 0.34±0.16, nPE: 0.46±0.37) maxima observed over the lateral prefrontal cortex. The statistical analysis revealed significant differences between groups exclusively in the theta band, patients with SLPE exhibiting significantly increased current density in the theta frequency band in the medial and lateral parietal cortex bilaterally, with the highest significance in the left medial parietal cortex (i.e., posterior cingulate/precuneus cortex) (SLPE: 0.30±0.32, nPE: 0.08±0.05, *t*_max_ = 3.08, p = 0.006) (Fig. 2).

### Functional connectivity

**Figure 3:**
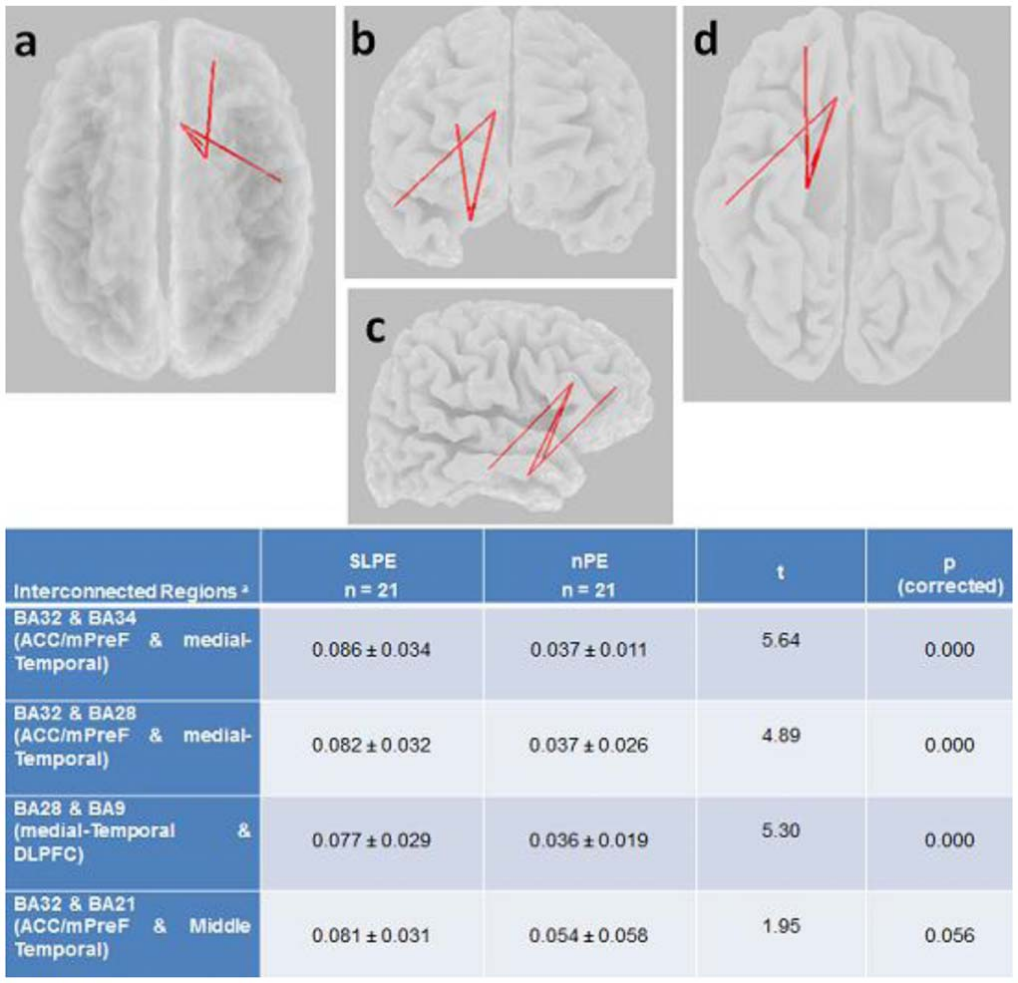
eLORETA wire diagram illustrating significantly increased functional connectivity (*lagged* phase synchronization) in the beta2 frequency band in right frontotemporal circuits in patients with schizophrenia-like psychosis of epilepsy vs. those with nonpsychotic epilepsy (*t*_max_ = 5.6; p<0.01, corrected). The significant connectivity wires are shown inside a transparent cortical surface with axial views from the top (left) and bottom (right), as well as coronal (top middle) and right sagittal view (bottom middle). The red color of the wire indicates relative increase in phase synchronization (as opposed to a blue wire that would indicate connectivity decrease). Increased beta2 (21–30 Hz) phase synchronization was observed between signals of the Brodmann area (BA) 32 (anterior cingulate/medial prefrontal cortex) and those of BAs 32/28 (medial temporal cortex) and BA21 (middle temporal gyrus in the lateral convexity), and between BA 32 and BA 9 (dorsolateral prefrontal cortex). The points to which the lines are connected represent the center of mass of the BAs. The table at the bottom shows the phase synchronization values of the significantly different connections between groups. ^a^ Regions in the right hemisphere; SLPE, schizophrenia-like psychosis of epilepsy; nPE, nonpychotic epilepsy; BA, Brodmann area; ACC, anterior cingulate cortex; mPreF, medial prefrontal; DLPFC, dorsolateral prefrontal cortex.

We tested for phase synchronization differences between SLPE and nPE groups in all frequency bands. The statistical eLORETA analysis indicated that patients with SLPE had significantly increased phase synchronization specifically in the beta2 (21–30 Hz) frequency band compared with those with nPE (Fig. 3). This connectivity was found in the right hemisphere between the medial temporal cortex (BA 28 and 34) and both the anterior cingulate cortex (ACC)/medial prefrontal cortex (mPFC) (BA 32) and the dorsolateral prefrontal cortex (BA 9), with the former showing the highest connectivity. In addition, the right lateral temporal cortex over the medial temporal gyrus (BA 21) also showed increased connectivity with the ipsilateral ACC/mPFC (BA 32) that very nearly reached statistical significance (corrected p = 0.056). The table in Figure 3 shows the significant difference in lagged phase synchronization values between groups.

### Correlation with clinical variables

**Figure 4:**
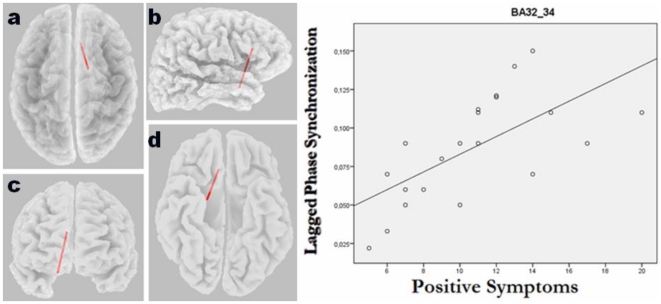
eLORETA wire diagram and scatterplots of significant correlations between functional connectivity (*lagged* phase synchronization values) in beta2 (21–30 Hz) frequency band and psychopathology scores (i.e., positive symptoms) in patients with schizophrenia-like psychosis of epilepsy at specific regions (r = 0.79; p<0.05, corrected). The significant connectivity wire is projected onto a transparent fiducial cortical surface. a) axial view from the top; b) sagittal view from the right; c) coronal view from the front d) axial view from the base. The points to which the lines are connected represent the center of mass of the BAs. BA32_34, connectivity between Brodmann areas 32 and 34.

**Figure 5:**
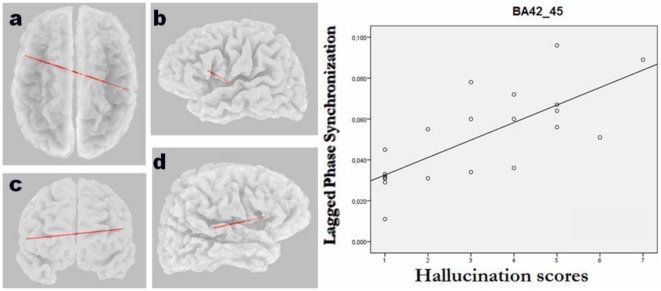
eLORETA wire diagram and scatterplots showing a significant correlation between interhemispheric beta2 (21-30 Hz) phase synchronization of right auditory cortex and the Broca's area with auditory hallucination scores among patients with schizophrenia-like psychosis of epilepsy (r = 0.82; p<0.05, corrected). The significant connectivity wire is projected onto a transparent fiducial cortical surface. a) axial view from the top; b) sagittal view from the left; c) coronal view from the front d) sagittal view from the right. The points to which the lines are connected represent the center of mass of the BAs. BA 42_45, connectivity between Brodmann areas 42 and 45.

The eLORETA correlation analyses revealed that the significantly increased lagged phase synchronization between the medial temporal cortex (BA 34) and the ACC/mPFC (BA 32) found in patients with SLPE relative to the nonpsychotic group correlated with positive symptoms (r = 0.79, p = 0.047, corrected). In addition, we found that among psychotic patients, “auditory” hallucination scores showed a significant correlation with increased interhemispheric beta2 lagged phase synchronization between the right auditory cortex (BA 42) and the Broca's area (BA 45) (r = 0.82, p = 0.032, corrected). Scatterplots for the significant correlations are given in Fig. 4 and 5. No correlation was found between connectivity measures and chlorpromazine equivalent dose of antipsychotic medication (r = 0.26, p = 0.71).

## Discussion

In the present study, we used eLORETA to determine differences in source current density and functional connectivity in patients with SLPE compared with those with nPE during resting state. A novel measure of nonlinear connectivity, namely lagged phase synchronization was used for analyses. Our findings revealed increased theta oscillations in the medial and lateral parietal cortex bilaterally in the psychotic epilepsy patients relative to their nonpsychotic counterparts. In addition, they had increased temporo-prefrontal connectivity in the beta2 frequency band, specifically in the right hemisphere. Furthermore, this increased connectivity in temporo-frontal circuits correlated with positive symptom scores, and there was increased interhemispheric phase synchronization between the auditory cortex of the affected temporal lobe and the Broca's area that correlated with hallucination scores.

Based on reports of low-frequency (i.e., delta, theta) resting EEG abnormalities related to disorder-specific pathophysiology in schizophrenia, the increase in slow oscillatory activity in the theta range found in this study most likely indicates resting-state cortical dysfunction in SLPE [61]. This activity was topographically distributed over regions in the medial (i.e., posterior cingulate/precuneus) and lateral parietal cortex that are part of the DMN, which is typically activated during rest [19]. Thus, our source-localization finding supports and extends the notion that the parietal lobe plays a crucial role in the pathogenesis of psychosis [20], [62], and that DMN dysfunction may be a core neurobiological feature in both schizophrenia [3], [5] and SLPE. Consistent with this argument, we previously reported abnormal parietal activation closely associated with the delusional state of patients suffering from episodic interictal epileptic psychosis [63]. In addition, Sundram et al [64] in a voxel-based morphometry study noted that extratemporal cortical abnormalities, in particular prominent parietal gray matter deficits occurred in patients with temporal epilepsy with psychosis. This evidence suggests that parietal dysfunction is not exclusive of the classic form of schizophrenia but may be also critical to the pathophysiology of schizophrenia-like disorders. It is noteworthy, however, that resting-state dysfuntion of the DMN, including the medial and lateral parietal cortex, can be seen in Alzheimeŕs disease, attention deficit hyperactivity disorder, and autism, which indicates that it may be a common feature of several chronic mental illnesses [65], [66].

Although there is increasing interest in connectivity deficits in schizophrenia, to our knowledge, this is the first study to explore functional connectivity in patients with psychosis in epilepsy. Most studies in schizophrenia indicate significant functional disconnection or reduced connectivity between wide brain areas as a possible pathophysiological mechanism underlying key deficits of the disorder [4]. These results come mainly from studies using fMRI, especially during performance of cognitive tasks in both first episode schizophrenia and the chronic state of the illness. Given the complexity of the disease, it is not surprising that in addition to functional disconnection, increased connectivity between certain brain areas is also observed to a lesser or greater extent in schizophrenia, sometimes representing the predominant pattern of aberrant connectivity [5]. This is particularly true for patients in an early stage of the illness during rest [4], and even in first degree relatives who are at increased risk of developing psychosis [5], [67], [68]. Based on this argument, it is interesting that compared to nonpsychotic patients we found a pattern of temporo-prefrontal hyperconnectivity specifically during resting state in patients with SLPE who are in a relatively early stage of the psychotic disorder.

Further evidence for a role of resting-state increased connectivity in some circuits as a core neural activity associated with psychotic symptoms comes from a recent fMRI pharmacological study demonstrating that antipsychotic-induced reduction of baseline functional hyperconnectivity in drug-naïve first episode schizophrenia correlated with clinical recovery of the patients [69]. Altogether, these findings suggest that a pattern of hyperconnectivity between certain brain regions may be an indicator of a vulnerability to psychosis and an early abnormality in both schizophrenia and the schizophrenia-like disorder occurring in epilepsy. It is noteworthy that although most studies looking at functional connectivity in psychoses [3]–[5], [20], [22], [70] and in epilepsy syndromes [71], [72] have used fMRI, the few existing EEG data on brain connectivity during rest in schizophrenia indicate that psychosis-related abnormal functional connectivity between cortical areas often occur in faster oscillations, particularly in the beta frequency band [73]. This supports our findings of altered beta2-phase synchronization in patients with SLPE.

The functional hyperconnectivity found in patients with SLPE relative to those with nPE in this study involved specifically temporal and prefrontal connections. In line with this observation, a recent review of structural and functional connectivity by Pettersson *et al.* highlighted that although there are no clear and unequivocal network alterations in terms of connectivity in schizophrenia, but instead a vast array of subtly altered networks throughout the brain, connectivity of frontal regions appears to be particularly compromised in this disorder [4]. Fletcher *et al.* using PET demonstrated that psychosis in schizophrenia was associated with abnormal temporo-prefrontal integration or with a disruption of the normal anterior cingulate modulation of this activity [70], which provides further support to our connectivity findings. Interestingly, the increase in lagged phase synchronization between temporal and prefrontal regions in our study affected specifically the right hemisphere. It might suggest a particular role for right-sided functional abnormalities in schizophrenia-like disorders, and in particular in SLPE. Alternatively, this might be related to the fact that the majority of the patients with SLPE in this study had mesiotemporal epilepsy with predominant right seizure focus (63.2% of the patients had right temporal epilepsy). This observation gives rise to the speculation that resting-state functional hyperconnectivity between irritative areas subserving psychic symptoms (e.g., medial temporal cortex) and cortical regions involved in the DMN (e.g., ACC/mPFC) [19], [20] or in neuropsychological models of psychosis (e.g., DLPFC) [3], [6], [64]–[66] might be a mechanism underlying chronic psychosis in patients with epilepsy. This does not suggest a synchronized “epileptic” activity and warrants further investigation. The fact that pronounced abnormalities (e.g., slowing) were not observed in the temporal lobe supports the idea that an abnormal functional connection of this region with areas relevant to psychosis rather than a local cortical dysfunction may be a key factor in SLPE pathophysiology.

A striking finding of our analyses is that the significantly increased lagged phase synchronization between the medial temporal cortex and prefrontal regions (i.e., ACC/mPFC) in patients with SLPE showed a positive correlation with the severity of psychopathological disturbance, in particular with positive symptoms. This speaks in favor of a strong relationship of the observed connectivity deficits with psychosis and SLPE pathophysiology. We also noticed that increased synchronization of the auditory cortex of the right hemisphere (where between-group connectivity deficits were found) with the Broca's area correlated with auditory hallucination scores. This result indicates functional connectivity of frontotemporal and language-auditory networks in patients with SLPE, which is consistent with fMRI reports demonstrating that functional connectivity of frontal and auditory cortex correlates with the severity of positive symptoms, including auditory hallucinations, as measured with the Positive and Negative Syndrome Scale (PANSS) [6]. Furthermore, this supports findings of previous studies proposing that the experience of auditory hallucinations is linked to increased activity in a frontotemporal network involved in speech generation (i.e., Brocás area) and speech perception (i.e., Wernickés area or auditory cortex) [74], [75]. Moreover, the evidence associating auditory hallucinations, the sensory auditory cortex, and lagged phase synchronization between this cortical region and language-generation areas in the left inferior frontal cortex can contribute to validate the eLORETA method in terms of nonlinear connectivity, thus supporting the feasibility of using this algorithm to detect functional connectivity in the normal and pathological brain.

It is important to emphasize that our results reflect genuine physiological connectivity since, unlike previously published methods, eLORETA algorithm is based on a proper model for the two components of a connection: instantaneous and lagged. In our experience, the connectivity patterns of both the classic phase synchronization and classic coherence (which include the instantaneous artifact) are dominated simply by the locations of activity maxima, which are seen as common instantaneous sources, and therefore are not related to true physiological connections [30], [31]. Compared to the imaginary part of the coherence proposed by Nolte et al. [27] as a conservative index of connectivity, the lagged connectivity measure has the important property of being relatively robust to the strength of the instantaneous component, i.e. it can still detect physiological “non-zero” lagged connectivity even in the presence of large instantaneous artifacts, while the imaginary part of the coherence fails to detect a lagged connection by tending to zero if the instantaneous component is large [30].This would also apply to phase synchronization, since it is nothing more than the coherence for unit modulus (amplitude free) Fourier transform coefficients. The phase lag index proposed by Stam et al. [26] represents an improvement over the imaginary part of the coherence because it is less affected by phase delay. However, it is also relatively insensitive to true changes in phase synchronization when the phase lies very close to zero, which is likely to happen as the instantaneous component increases. Thus, it is possible that the specific physiological lagged connections observed in our study might have not been detected by the classic synchronization method and other versions that attempt to correct for the artifactual instantaneous component.

Our results should be interpreted with caution based on the limitation of a relatively small size and the possible confounding effect of antipsychotic medication. However, a small sample is not uncommon in neuroimaging research on SLPE, which is thought to be the direct result of the difficulty in recruiting patients with SLPE and single brain pathology owing to the rarity of the disorder. Nevertheless, we matched the patient groups for age and epilepsy characteristics, and those with low IQ and gross organic lesions were not included. Most patients in the psychotic group were taking low dose of antipsychotics (mean chlorpromazine equivalent dose of 203.7±308.4 mg/day). However, there was no significant correlation between chlorpromazine equivalent dose and connectivity measures, suggesting that pharmacological agents may not influence our results. Another potential limitation is that our ROI-based connectivity analysis relied on cytoarchitectonically-defined regions (i.e., BAs) that are projected onto the Talairach atlas and show interindividual variablity due to differences in brain size, shape, etc [76], [77]. Although using a BA-approach to explore the convergence of brain function and structure is a common practice in neuroimaging studies, a perfect specification of cortical regions is not guaranteed, and the possibility that this might affect estimates of functional connectivity cannot be ruled out.

Overall findings indicate that in addition to dysfunction of parietal regions that are part of the DMN, resting-state disrupted connectivity of the medial temporal cortex with prefrontal areas that are also involved in the DMN or implicated in psychopathological dysfunction may be critical to schizophrenia-like psychosis, especially in individuals with temporal lobe epilepsy. This suggests that DMN dysfunction may be a core neurobiological feature of the disorder. Increase in theta oscillations and in beta phase synchronization appears to represent the underlying neural activity of these deficits. These findings warrant further investigation using larger samples of drug-naïve patients with epilepsy psychosis and eLORETA connectivity method.
